# The potential of gypsum speleothems for paleoclimatology: application to the Iberian Roman Human Period

**DOI:** 10.1038/s41598-020-71679-3

**Published:** 2020-09-09

**Authors:** Fernando Gázquez, Thomas K. Bauska, Laia Comas-Bru, Bassam Ghaleb, José-María Calaforra, David A. Hodell

**Affiliations:** 1 Water Resources and Environmental Geology Research Group, Department of Biology and Geology, University of Almería, Crta. Sacramento s/n, La Cañada de San Urbano, 04120 Almería, Spain; 2 Andalusian Centre for the Monitoring and Assessment of Global Change (CAESCG), University of Almería, Crta. Sacramento s/n, La Cañada de San Urbano, 04120 Almería, Spain; 3 Godwin Laboratory for Palaeoclimate Research, Department of Earth Sciences, University of Cambridge, Downing Street, Cambridge, CB2 3EQ UK; 4 British Antarctic Survey, High Cross, Madingley Road, Cambridge, CB3 0ET UK; 5 School of Archaeology, Geography & Environmental Sciences, University of Reading, Whiteknights, Reading, Berkshire, RG6 6DR UK; 6 Centre de Recherche en Géochimie Et Géodynamique (GÉOTOP-UQAM)-McGill University, 201 ave. du Président-Kennedy 7e étage, local PK-7150, Montréal, QC H2X 3Y7 Canada

**Keywords:** Geochemistry, Palaeoclimate

## Abstract

Carbonate cave deposits (speleothems) have been used widely for paleoclimate reconstructions; however, few studies have examined the utility of other speleothem-forming minerals for this purpose. Here we demonstrate for the first time that stable isotopes (δ^17^O, δ^18^O and δD) of structurally-bound gypsum (CaSO_4_·2H_2_O) hydration water (GHW) can be used to infer paleoclimate. Specifically, we used a 63 cm-long gypsum stalactite from Sima Blanca Cave to reconstruct the climate history of SE Spain from ~ 800 BCE to ~ 800 CE. The gypsum stalactite indicates wet conditions in the cave and humid climate from ~ 200 BCE to 100 CE, at the time of the Roman Empire apogee in Hispania. From ~ 100 CE to ~ 600 CE, evaporation in the cave increased in response to regional aridification that peaked at ~ 500–600 CE, roughly coinciding with the transition between the Iberian Roman Humid Period and the Migration Period. Our record agrees with most Mediterranean and Iberian paleoclimate archives, demonstrating that stable isotopes of GHW in subaerial gypsum speleothems are a useful tool for paleoclimate reconstructions.

## Introduction

Carbonate speleothems (i.e. calcite and aragonite stalagmites and flowstones) are important archives for Quaternary climate reconstructions in terrestrial environments^1,2^. In addition to calcite and aragonite, over 300 other minerals have been reported from caves^3^. After carbonates, sulfates are the second most important group with gypsum (CaSO_4_·2H_2_O) being the most common sulfate mineral in caves^4,5^.

Subaerial gypsum speleothems can form during the evaporation of calcium-sulfate-rich solutions in subterranean environments. The dissolution of gypsum host-rock (e.g. Messinian marine gypsum) supplies Ca^2+^ and SO_4_^2−^ to cave dripwater and subsequent in-cave evaporation leads to a solution that is saturated with respect to gypsum^5^. Gypsum speleothem formation is mostly controlled by evaporation, unlike carbonate speleothems that largely form because of CO_2_ degassing. Although the occurrence of subaerial gypsum speleothems is limited compared to carbonate speleothems^3^, gypsum stalactites, stalagmites, flowstones, coralloids and frostwork can be found in a number of caves in arid and semiarid regions^5–7^.

The isotopic composition of evaporated dripwaters (i.e. δ^17^O, δ^18^O and δD, and derived parameters d-excess and ^17^O_excess_; see “Methods” for definitions) in caves of semiarid environments is recorded by the structurally-bound Gypsum Hydration Water (GHW) at the time of gypsum speleothem formation, which is mainly driven by evaporation^5^. The fractionation factor between GHW and the mother solution (dripwater) is relatively insensitive to temperature^8,9^. This contrasts with other isotopic proxies in speleothems (e.g. δ^18^O in carbonates) that can be influenced by temperature and kinetic effects. Our recent ability to measure all the stable isotopes (^16^O, ^17^O, ^18^O, ^1^H, ^2^H) of GHW^10^ is advantageous for using GHW as a paleoclimate archive. Indeed, recent investigations have utilized triple oxygen and hydrogen isotopes of GHW from marine and lacustrine gypsum deposits to reconstruct the climate of the past^11–15^.

**Figure 1:**
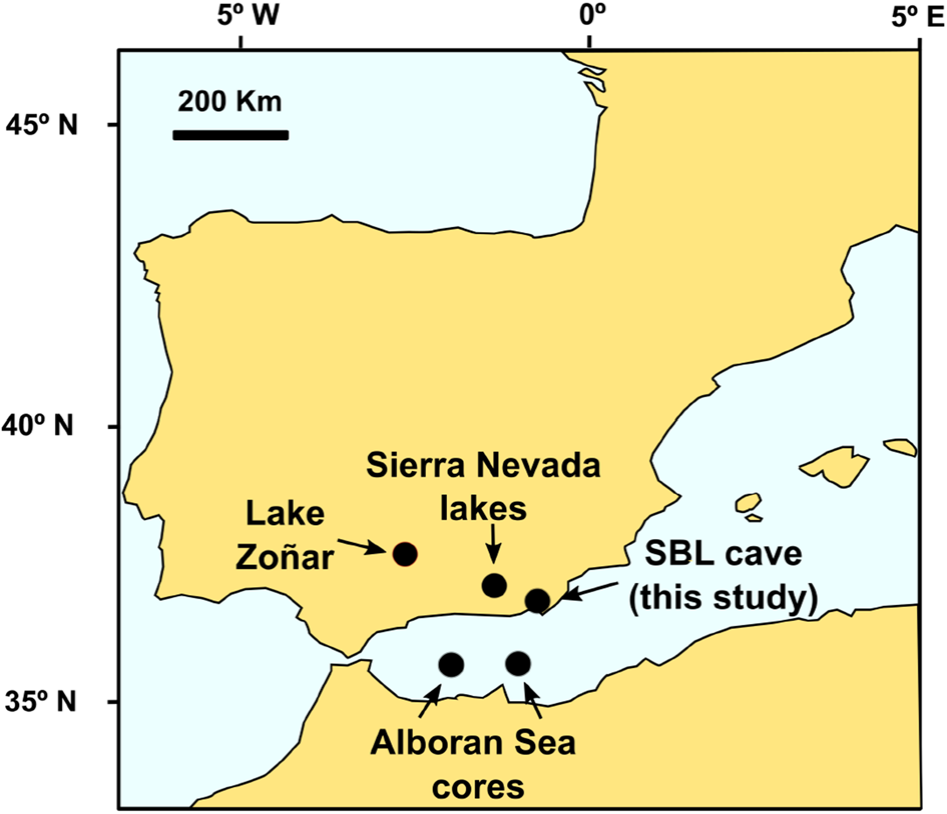
Main regional paleoclimate records mentioned in this study. This includes Sima Blanca cave (SBL cave, this study); Lake Zoñar^30–33^; alpine lakes of Sierra Nevada^26,34–38,41,47^ and Alboran Sea sediment cores, ODP Site 976 and 300G^32^. Figure created by InkScape 0.92.4 (https://inkscape.org).

The Sorbas Basin (SE Spain) is a type locality for gypsum karst and has a well-developed system of caves with varying degrees of decoration^5^. Here we use a 63 cm-long gypsum stalactite (SBL hereafter) from Sima Blanca Cave (402 m a.s.l) (Fig. 1 and Supplementary Fig. S1 online) to reconstruct the isotopic composition (δ^17^O, δ^18^O and δD, and derived ^17^O_excess_ and d-excess) of evaporated cave dripwater from ~ 2.800 to ~ 1.200 years BP. This period spans the Iron Age (800–650 BCE), the Iberian Roman Humid Period (IRHP; 650 BCE–476 CE) and the Migration Period (476–800 CE). Throughout this study, “years BP” refers to years before present, where present is 1950 of the Common Era (CE).

Previous studies have suggested that the fate of various civilizations occupying the Andalusian lowlands (southern Iberia Peninsula) may have been affected by climatic change, which impacted the availability of freshwater and agriculture^16^. Among the different ancient civilizations that the occupied Andalusia, the Roman Empire (~ 200 BCE to ~ 400 CE) left a rich cultural and architectural heritage in the Roman Iberian province of Hispania Ulterior (197 BCE to 27 CE), later called Baetica (27–411 CE)^17^. Most archaeological and historical reconstructions agree that the abandonment of the Iberian Peninsula by the Romans was mainly caused by a generalized sociopolitical collapse, together with the Barbarian invasions that contributed to the fall of the whole Roman Empire^18^. However, various paleoclimate reconstructions also suggest that climate change may have played a role, including the occupation and the abandonment of the Roman Iberian province of Baetica^16^. Independent written and archaeological evidence consistently support variable climate conditions during the period of the Roman Empire’s expansion and final demise.

Yet, terrestrial paleoclimate reconstructions from the region are scarce. We aim to establish if the stable isotope ratios of hydration water in gypsum speleothems can be used as a paleoclimate proxy to reconstruct past changes in wetness/dryness of SE Iberia between 800 BCE and 800 CE. The Sima Blanca stalactite grew during the period of Roman occupation, and the paleoclimate history inferred from its GHW is compared to other Mediterranean and Iberian paleoclimate archives to evaluate the reliability of gypsum speleothems as a recorder of past climate conditions (Fig. 1).

## Results

### U-Th dating

Details of sampling and labeling are given in the “Methods” section and in the “Supplementary Materials”. The inner structure of gypsum stalactites is more complex than that of conventional carbonate stalagmites used for paleoclimate purposes (Supplementary Fig. S2 online). We avoided sampling near the SBL’s inner channel that, in places, contains microcrystalline gypsum that likely precipitated during the late stage of formation of the speleothem before growth stopped.

The U and Th contents in SBL stalactite are relatively low, as in most gypsum speleothems analysed previously^19^. The ^238^U content in the dated gypsum samples ranges from 94 to 178 ppb. Low U content and thus extremely low ^230^Th concentration precluded obtaining an age for sample SBL-33. As ^230^Th/^232^Th is < 20 (5.1–14), a correction for ^230^Th contamination was needed to obtain accurate ages (see “Methods”). The ^230^Th-corrected ages differ by ~ 200 years from the uncorrected values (Supplementary Table S1 online). Age errors are given as ± 2σ and vary from 6 to 12%. These errors are comparable to those obtained for carbonate speleothems of similar ages with low U concentrations (i.e. < 200 ppb)^20^. The relatively high content of detrital thorium (^232^Th) in the SBL stalactite is probably related to clay minerals from the Messinian host-rock (i.e. gypsum-marl beds) or detrital contributions from the soil above the cave. Low U concentrations and high detrital Th of gypsum speleothem hinder extremely accurate and precise dating of gypsum speleothems in the Sorbas basin, but the errors are sufficiently low (± 129 to ± 334 years) to permit comparison with other regional paleoclimate records.

**Figure 2:**
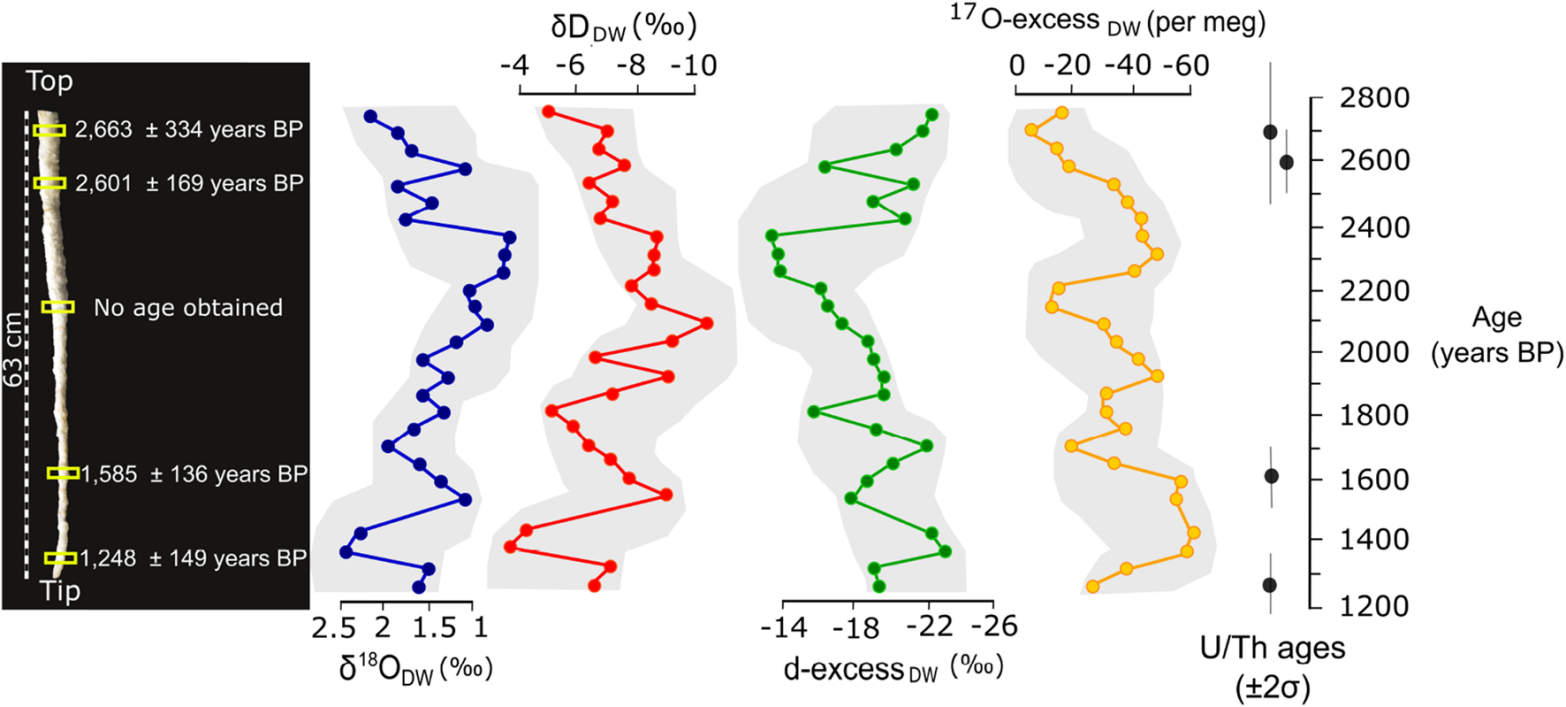
U–Th ages of the SBL stalactite (see Supplementary Table S1 online for details) and triple oxygen and hydrogen isotope reconstruction of speleothem-forming water in Sima Blanca cave. The isotopic composition of evaporated paleo-dripwater (DW) are obtained from GHW (Supplementary Table S2 online) using the fractionation factors between free water and GHW^8,9^. The grey shades represent the time-series 95% confidence interval of the proxies obtained from COPRA^21^ which combines analytical and chronological errors.

SBL stalactite grew from 2,666 ± 334 to 1,248 ± 149 years BP with no evident growth hiatuses. The lack of age reversals in our U/Th ages suggests that the sample sequence taken from the gypsum stalactite is in stratigraphic order. The COPRA-based age-depth model^21^ is anchored by four U-Th ages (Supplementary Table S1 and Fig. S3 online and Fig. 2) and indicates a near linear growth rate of 0.42 mm/year. The average temporal resolution of the stable isotope record of GHW is ~ 60 years.

### Stables isotopes in gypsum hydration water

The δ^17^O of GHW ranges from 2.1 to 3.0‰, δ^18^O varies from 4.1 to 6.0‰, and δD ranges from − 30.7 to − 23.7‰ (Supplementary Table S2 online). The isotope fractionation factors between free (mother) water and GHW at the mean cave temperature of 15 °C are^8,9^: α^17^O_gypsum–water_ = 1.00188; α^18^O_gypsum–water_ = 1.00354 and αD_gypsum–water_ = 0.979. We used these to calculate the isotopic composition of the water from which the gypsum was precipitated, which we hereafter refer to as the speleothem-forming water. These fractionation factors are not affected by temperature in the range of the expected cave temperatures during SBL stalactite growth (i.e. 12–16 °C) and thus can be assumed to be constant. For instance, the use of temperatures that are 10 °C higher or lower changes the δ^18^O values by only ~  ± 0.05‰ and δD by ~  ± 1‰, which is not very significant relative to the analytical precision of our method.

**Figure 3:**
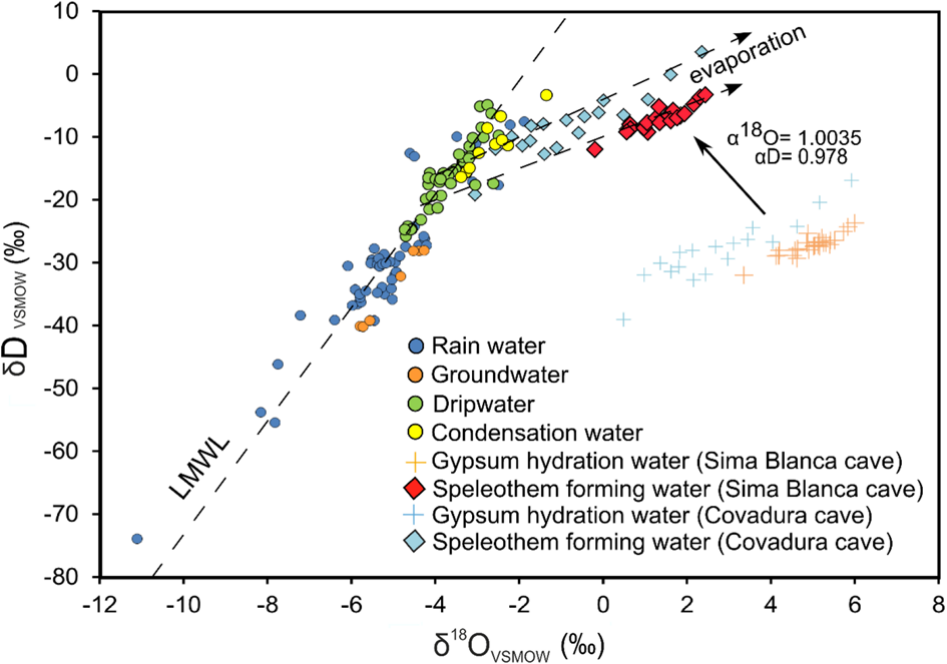
δ^18^O vs δD (V-SMOW) of speleothem-forming waters in Sima Blanca cave, inferred from GHW in the SBL stalactite, after correction with fractionation factors (see main text). Speleothems lie on an evaporation line that intersects the isotopic composition of dripwater (a mixture of infiltration and condensation water) in these caves. Values of rainwater and groundwater in the Sorbas region and dripwater and other gypsum speleothem-forming waters in Covadura cave (2 km apart from Sima Blanca)^5^ are displayed for comparison. The expression of the Local Meteoric Water Line (LMWL) is δD = 7.2 δ^18^O + 7.2^5^.

**Figure 4:**
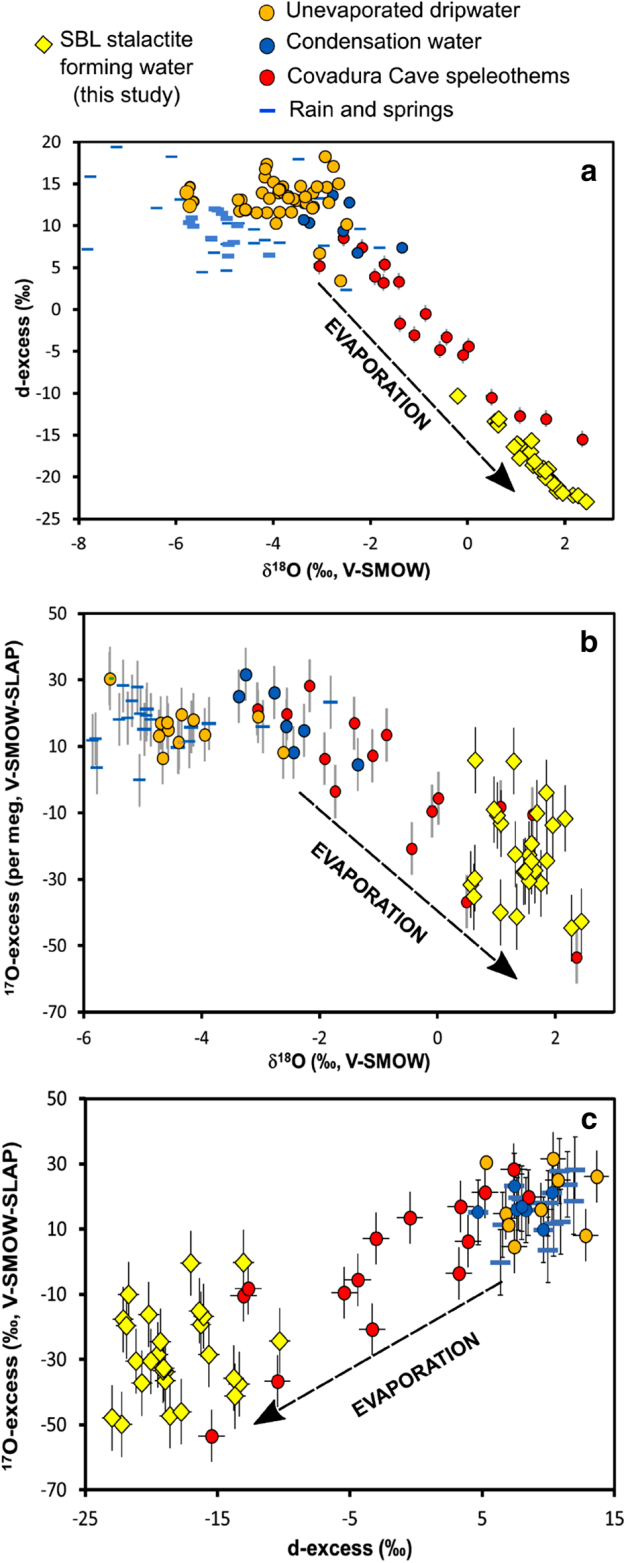
Cross-plots of stable isotopes in gypsum speleothem-forming water (obtained from GHW after applying fractionation factors; see main text) in Sima Blanca Cave, compared to gypsum speleothem-forming water in Covadura Cave (2 km apart from Sima Blanca), unevaporated cave water (dripwater and condensation water) and rainwater in the gypsum karst of Sorbas^5^.

The δ^17^O, δ^18^O and δD of the speleothem-forming water show the lowest values (0.3, 0.6 and − 10.7‰, respectively) at ~ 2,100 years BP, whereas the highest values (1.2, 2.4 and − 3.4‰, respectively) occur at ~ 1,400 years BP (Fig. 2). The d-excess of the speleothem-forming water ranges from − 22.9 to − 13.4‰, with a sharp increase of ~ 7‰ at ~ 2,300–2,200 years BP. The ^17^O_excess_ of the speleothem-forming water ranges from − 51 to − 10 per meg (Fig. 2) with its absolute minimum and maximum values at ~ 2,700 and 1,400 years BP, respectively. The δ^18^O–δD values of the speleothem-forming water are linearly correlated (Fig. 3), with a slope of 2.9 (R^2^ = 0.72) that is considerably lower than that of the Local Meteoric Water Line (LMWL) (Fig. 3), indicating the dripwater was evaporated before reaching gypsum saturation. The ^17^O_exccess_ and d-excess are negatively correlated with δ^18^O (Fig. 4).

## Discussion

### Mechanisms controlling gypsum speleothem formation and the isotopic composition of GHW

Previous studies on subterranean microclimate parameters in the gypsum karst of Sorbas Basin (i.e. Covadura Cave, 2 km apart from Sima Blanca Cave) concluded that the precipitation of gypsum speleothems is favoured during the winter dry season (January–March), when relative humidity in the upper cave passages is less than 95% and occasionally below 60%^5^. Low winter cave relative humidity results from an intense exchange of air masses with the external atmosphere when the outside air temperature falls below the cave’s air temperature^5^. From April to December, relative humidity in the cave’s atmosphere is closer to water saturation (normally above 95%), decreasing evaporation in the cave and thus suppressing gypsum precipitation. Consequently, as evaporative conditions are more likely to occur during winter, we interpret the isotopic record of the SBL stalactite as reflecting winter evaporative conditions in the cave.

In addition to infiltration water, condensation of atmospheric water vapour has been found to be an important source of water for gypsum speleothem formation during summer and autumn^5,22^. Water condenses on the cave ceiling, dissolving Messinian gypsum host-rock and the water becomes saturated with respect to gypsum. The saturated water then migrates by gravity along the surface of the stalactite, mixing along its path with seepage water, and gypsum deposition occurs because of evaporation. The relative contribution of the condensation water source to the dripwater was estimated to be ~ 40% in the nearby Covadura Cave system. The intensity of this condensation process depends on the difference between external and internal cave temperatures and on the degree of cave ventilation^5,22^. Sima Blanca cave is a shallow subterranean system (~ 10 m deep) and its temperature is expected to equilibrate rapidly with the external atmospheric temperature. This has been observed in the passages of Covadura Cave system (also ~ 10 m deep), where the equilibration time is < 2 months^23^. As a result, we do not expect the condensation to seepage water ratio to change significantly either on an inter-annual basis or during the growth of the SBL stalactite. In addition, the modern mean isotopic composition of dripwater and condensation water in these caves differ by only ~ 1‰ for δ^18^O and ~ 5.5‰ for δD, whereas their d-excess and ^17^O_excess_ are indistinguishable within error^5^. This reinforces the idea that long-term changes in the condensation to seepage water ratio did not significantly alter the isotopic composition of the SBL stalactite, but rather isotopic changes mainly reflect the degree of evaporation within the cave.

Once meteoric water reaches the cave and mixes with condensation water, the most important process affecting δ^17^O, δ^18^O and δD and derived parameters d-excess and ^17^O_excess_ is evaporation^5^. The absence of stalagmites in Sima Blanca suggests that most dripwater is evaporated from the speleothems on the ceiling. We observe that the values of d-excess and ^17^O_excess_ versus δ^18^O describe an evaporation trend (i.e. lower d-excess and ^17^O_excess_ values when δ^18^O increases) (Fig. 4). In general, the SBL speleothem-forming waters are more evaporated than modern cave dripwater (2011–2013 period)^5^ (Fig. 4). Higher infiltration/evaporation ratios are expected during wetter periods because of enhanced rainfall and higher relative humidity in the cave. This results in a lowering of δ^17^O, δ^18^O and δD values and higher d-excess and ^17^O_excess_ of the evaporated water^14,15,24^. During drier periods, the δ^17^O, δ^18^O and δD values of evaporated dripwater increase, whereas d-excess and ^17^O_excess_ decrease when aridity increases because of lower infiltration/evaporation.

Changes in the isotopic composition of rainfall and, therefore, infiltration water to the cave may also influence the SBL stalactite record. There are two main sources of humidity that may influence the isotopic composition of rainfall in the Mediterranean part of the Iberian Peninsula^25,26^. Convective rainfall events from the Mediterranean are isotopically enriched, and prevail during summer and autumn and during warm climatic periods. In contrast, rainfall with lower isotopic values are transported along Atlantic storm tracks, which dominate during winter and during colder climatic periods^25,26^ The mean δ^18^O values of modern rainfall from the Mediterranean have been found to be up to 3‰ higher in this region than rainfall events from Atlantic fronts^27^. Also, the d-excess values in rainfall from Mediterranean convective events are normally higher than in precipitation from the Atlantic moisture source^28^. Changes in the contributions of the Mediterranean and Atlantic moisture sources to the total hydrological budget in SE Iberia have been proposed previously to explain the δD variability of n-alkanes in sediment profiles of alpine lake of the Sierra Nevada^26^. This mechanism could have partially influenced the isotopic composition dripwater in Sima Blanca Cave; however, the fact that the δ^18^O–δD values of the speleothem-forming water are linearly correlated, with a slope much lower than that of the Local Meteoric Water Line (Fig. 3), strongly suggests that evaporation played a dominant role in controlling the isotopic variations of the dripwater recorded by the speleothem. Thus, we interpreted the long-term δ^18^O, δD and d-excess changes in the SBL stalactite dominantly as a record of evaporation in the cave that could have been affected by variations in the isotopic composition of rainfall in this region.

No specific studies have been conducted to measure the ^17^O_excess_ of past rainwater in this region. However, monitoring of triple oxygen isotopes of rainfall in southern Japan reported modern annual ^17^O_excess_ cyclicity (values ranging from ~ 10 to ~ 40 per meg) that is positively correlated to normalized relative humidity at the oceanic moisture source^29^. Water evaporation under low relative humidity conditions at the moisture source generates water vapour (and rainwaters) with greater ^17^O_excess_ values^30^. This contrasts with the behaviour of ^17^O_excess_ in evaporated waters that tends to be lower when evaporation occurs under low relative humidity conditions^14,15^. Therefore, the ^17^O_excess_ record of the SBL stalactite may be partially controlled by the long-term variability in the triple oxygen isotope composition of rainwater/infiltration water in the Sorbas area and the effects on ^17^O_excess_ of dripwater evaporation in the cave varying relative humidity conditions. Other potential mechanism may include changes in the main moisture source region (i.e. Mediterranean vs Atlantic source) or changes in the contributions of a non-rainfall water source (e.g. fogs) to cave dripwater.

In summary, the SBL stalactite isotope record is interpreted here as a function of infiltration/evaporation ratio in the cave, which is affected by rainfall amount in SE Iberia and humidity in Sima Blanca Cave. Also, varying contributions of the Atlantic and Mediterranean moisture sources to rainfall in this region could have influenced the isotopic signal of dripwater in the cave. Higher δ^17^O, δ^18^O and δD values derived from drier conditions and potentially from higher contributions of rainfall from the Mediterranean moisture source; lower δ^17^O, δ^18^O and δD, indicate wetter periods and may also suggest a greater influence of the Atlantic moisture source to the precipitation in this region.

Lower d-excess values in the SBL record indicate drier climate, although the signal may have been attenuated by higher d-excess of rainfall in response to greater contributions of Mediterranean convective precipitation events during dry periods. Higher d-excess values of GHW suggest wetter climatic conditions, with possible attenuation of the signal because of greater influence of the Atlantic rain events, which have lower d-excess values. The mechanisms controlling ^17^O_excess_ in GHW of gypsum speleothems in Sima Blanca Cave is more complicated, and there is very little information available for ^17^O_excess_ variability of modern rainfall in this region. Therefore, interpretation of the ^17^O_excess_ record of SBL remains speculative.

### The Sima Blanca paleoclimate record

**Figure 5:**
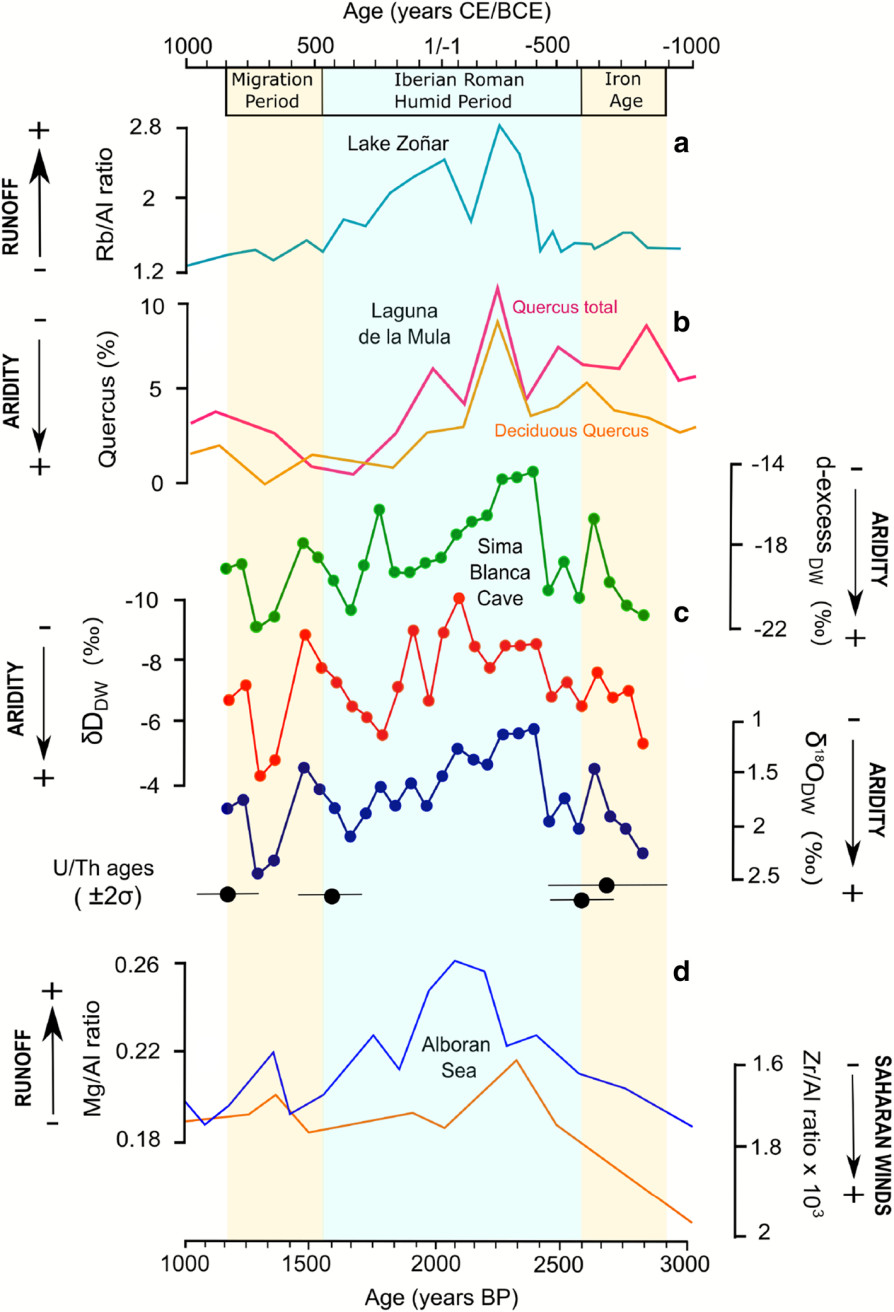
Comparison of SBL stalactite record with other local and regional paleoclimate proxies: (**a**) Rb/Al ratio in sediments of Lake Zoñar^33^; (**b**) *Quercus* pollen (%) in Laguna de la Mula^36,38^; (**c**) Reconstructed stable isotopes (δ^18^O, δD and d-excess; V-SMOW) of evaporated paleo-dripwater in Sima Blanca cave from the SBL gypsum stalactite. U/Th ages and errors are given below the record; (**d**) Mg/Al and Zr/Al ratios in Alboran Sea cores ODP 976 and 300G^33^.

The climatic changes inferred from the oxygen and hydrogen isotope ratios in GHW of the SBL stalactite are consistent with other nearby paleoclimate records from the region (Fig. 1), including Lake Zoñar in Andalucía^31–34^, several alpine lakes in Sierra Nevada^35–39^ and sediment records in the Alboran Sea^33^ (Fig. 5). Several lines of evidence suggest that the geochemical signal of the gypsum stalactite is primary and has not been altered by post-depositional processes (i.e. isotope exchange or gypsum dissolution/re-precipitation): (1) the SBL isotope record agrees with other regional paleoclimate proxies; (2) the reconstructed δ^18^O and δD of the speleothem-forming water describe an evaporation line with changing distance along the line indicating different degrees of evaporation; and, (3) there are no age reversals in the U/Th dates.

The SBL stalactite started growing at ~ 800 BCE. Dry conditions in southern Iberia prior to 800 BCE, as evidenced by a lower-than-average level of Lake Zoñar^31,32^, may have prevented stalactite formation. Likewise, low concentration of *Quercus* pollen in sediments of alpine lakes of Sierra Nevada are indicative of dry conditions during the Late Iron Age^37,38^ (Fig. 5). The relatively high δ^17^O, δ^18^O and δD values (1.3, 2.2 and − 5‰, respectively) of speleothem-forming water and relatively low d-excess (− 22‰) at 800 BCE compared to the mean value of the entire record (δ^17^O = 0.7‰, δ^18^O = 1.4‰, δD = − 7.2‰ and d-excess = − 18.1‰) indicates generally drier climate prevailed at this time. Also, greater contribution of the Mediterranean vapor source to rainfall in this region could result in higher isotopic values of dripwater in the cave during this period.

Consistent with a trend towards wetter conditions, δ^17^O, δ^18^O and δD values of evaporated paleo-dripwater decreased from 800 until ~ 500 BCE by about 0.5, 1.1 and 2.9‰, respectively, while d-excess increased by 5.9‰. This period includes the onset of the Iberian Roman Humid Period (IRHP), as inferred from the sediments of Lake Zoñar (650–510 BCE)^33^. A sharp decrease in δ^17^O, δ^18^O and δD values between 400 and 300 BCE result in the lowest isotopic values observed in the entire record. This abrupt change is accompanied by an increase in d-excess by ~ 8‰, until − 14‰, which is the highest d-excess value recorded in the SBL stalactite, indicating a trend toward wetter conditions. This shift in the isotope records and inferred wetter conditions coincide with an increase in Rb/Al recorded in the Lake Zoñar sediments at 400 BCE, as a result of enhanced runoff because of increased rainfall^33^. In addition, the concentration of *Quercus* pollen in Laguna de la Mula in the Sierra Nevada started to increase from 350 BCE as a result of enhanced water availability^36,38^.

The δ^17^O, δ^18^O, δD and d-excess values stabilized from 300 to 100 BCE at ~ 0.3‰, 0.6‰, − 8‰ and − 13‰, respectively, which are among the lowest values of the record (Fig. 5), suggesting wetter conditions that may have been accompanied by greater contributions of rainfall events from an Atlantic moisture source^26^. This wetter-than-average climate occurred around the time of the Roman occupation of Iberia (197 BCE) and is consistent with increased input of fluvial sediment to the Alboran Sea at that time^33^. Also, a large range of paleoclimatic records support these wet conditions in southern Iberia at ~ 300–200 BCE (Fig. 5) including enhanced runoff to Lake Zoñar^32,33^, an increase in deciduous *Quercus* pollen in Laguna de la Mula^36,38^; a decrease in Sahara dust input and increase in detrital sedimentation in Laguna de Hondera^35^; and enhanced aquatic production in the Borreguil de la Caldera and the Padul peat bog records^37,39^. Previous studies have identified this period as the wettest stage of the IRHP that began gradually from 650 BCE and reached the wettest conditions during the Iberian–Early Roman Epoch (510–190 BCE)^32^. The SBL record and other lake proxies in the region suggest this change may have been more abrupt.

Following this wet period, the SBL data support an aridification trend from ~ 100 BCE to 600 CE. This is indicated by the decrease in d-excess (− 10‰) during this period. The drying inferred from Sima Blanca cave agrees well with other Iberian terrestrial and marine records (Fig. 5) that document a general late-Holocene aridification from 5,000 years BP to present. This trend is also observed in alpine lakes of the Sierra Nevada^37–39^ and recurrent desiccation of other lakes in south Iberia^40,41^. The driest stage of the SBL record occurred at ~ 500 CE, when δ^17^O, δ^18^O, δD and d-excess of the evaporated paleo-dripwater reach their highest values (1.2‰, 2,4, − 3.4‰ and − 22.9‰). An increase in the frequency and intensity of convective rainfall events from the Mediterranean could have contributed to the relatively high δ^17^O, δ^18^O, δD values recorded by the speleothem during this period.

The latest part of the SBL record (from ~ 600 to ~ 800 CE) indicates conditions slightly wetter than during the previous 300 years, but drier than the period from 200 to 100 BCE. Dry conditions during the early stages of the Migration Period are indicated by a prominent decrease in Mediterranean forest in SE Iberian mountains, by an increase in *Cichorioideae* herbs and by the decline in the water levels in alpine lakes in the area^39^. Additionally, a progressive drying around 700 CE has been observed in Alboran Sea sediments. The SBL stalactite stopped growing at ~ 800 CE probably because of a reduction in water infiltration to the cave. This coincided with the last stages of the Visigoth occupation of Hispania (~ 800 CE), when generally dry conditions prevailed in Iberia^33,39,42^.

**Figure 6:**
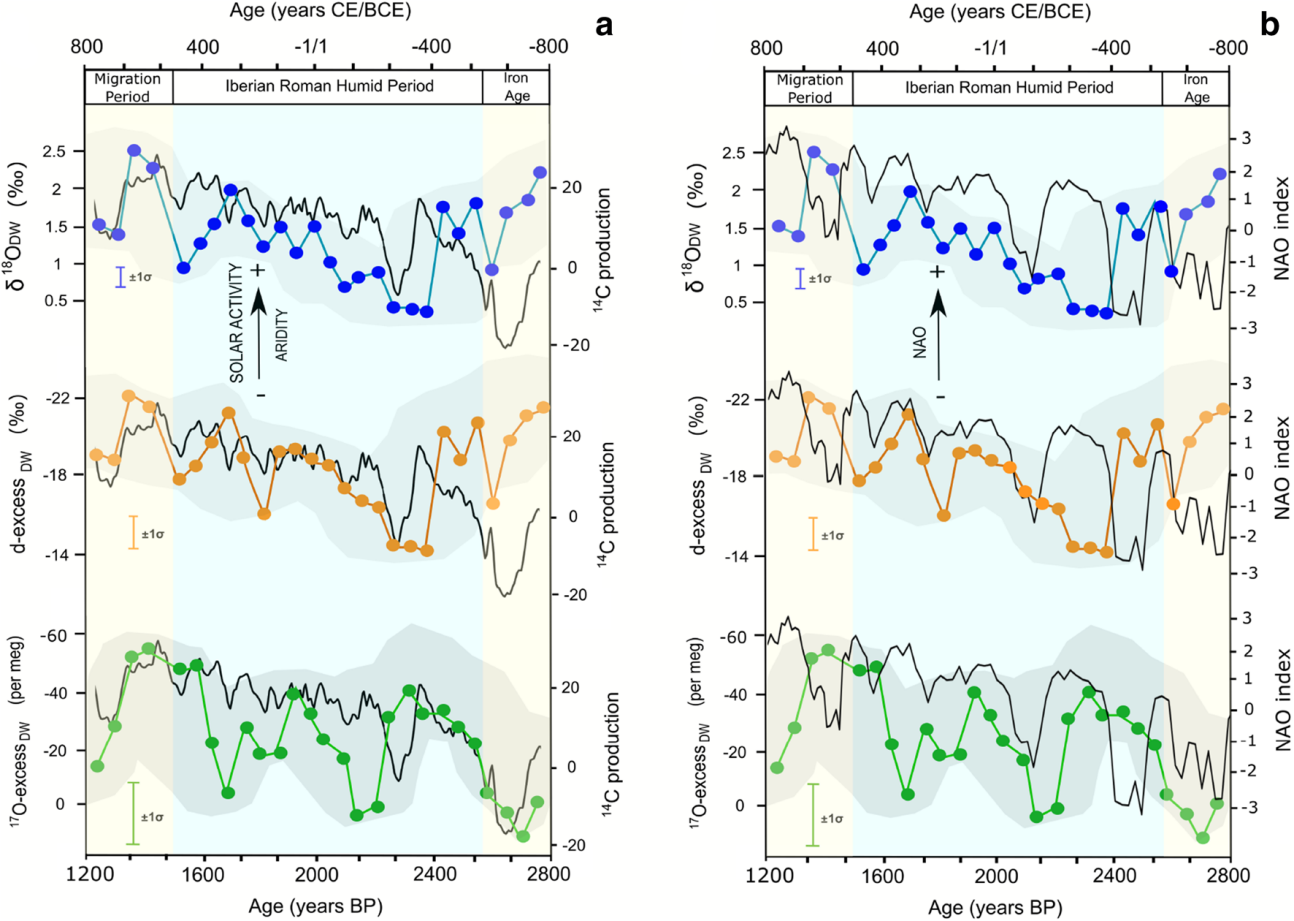
Stable isotope composition of paleo-dripwater in Sima Blanca cave, (**a**) the ^14^C atmospheric production as an indicator of solar activity^45^ and (**b**) the North Atlantic oscillation (NAO index)^44^. The grey shades represent the time-series 95% confidence interval of the proxies obtained by the COPRA code^21^ that combine analytical errors of stable isotope measurements and the age model errors.

Previous studies in Andalusia have suggested a possible link between varve thickness and geochemical proxies in several lakes of lowlands and alpine areas and the North Atlantic Oscillation (NAO) and solar variability^32,38,43^. The SBL stalactite record between ~ 800 BCE and ~ 800 CE also generally correlates with changes in the NAO (North Atlantic Oscillation) index^44^ and atmospheric radiocarbon production^45^. It is therefore possible that the aridification trend in SE Iberia may have been related to increased solar activity and NAO+ conditions (Fig. 6). While the ^17^O_excess_ record of SBL tracks the variability of solar activity and NAO throughout the record, variation in δ^18^O, δD and d-excess is anti-correlated during the Iron Age (800–500 BCE). Additional monitoring of modern conditions, including measurement of ^17^O_excess_ in dripwater and rainfall in the region may help to better understand the climate signal recorded by the SBL stalactite.

### Socio-cultural changes in south Iberia during the formation of the SBL stalactite

The wettest conditions inferred from the SBL stalactite (Fig. 6) occurred around the time of the Roman occupation of Iberia (197 BCE). Specifically, the wettest period starting at ~ 300–200 BCE agrees (within errors of the age model) with the time of the Second Punic Roman War (218–201 BCE) and the defeat of the Carthaginian by the Romans (206 BCE). The Roman Republic slowly expanded its control over Hispania over the next 200 years, which coincided with relatively wet climate in southern Spain that was probably favorable to cropping and farming.

**Figure 7:**
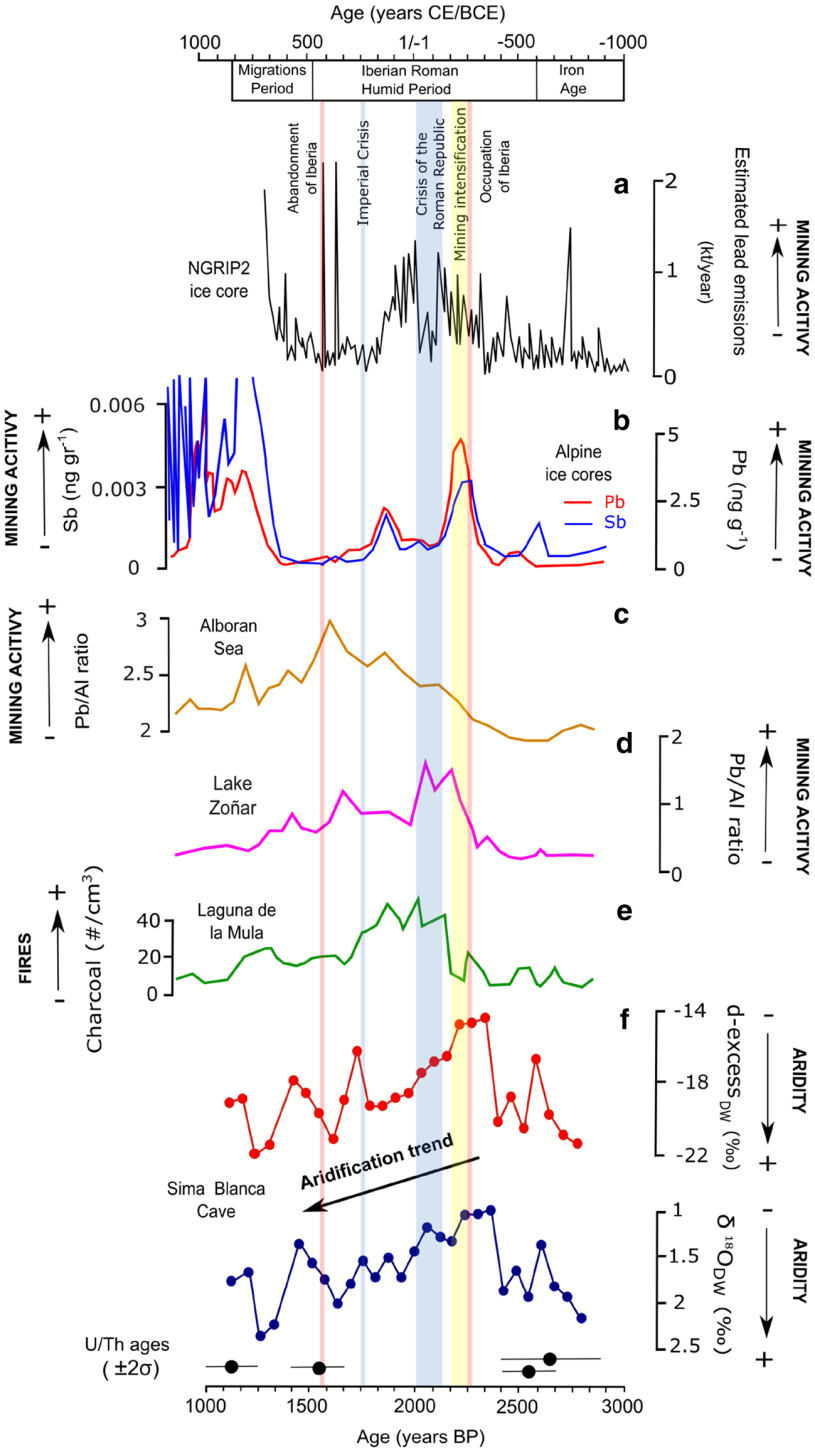
Pollution proxies from 3,000 to 1,000 years BP compared to the Sima Blanca paleo-humidity record: (**a**) Estimation of global lead emissions reconstructed from lead concentration in Greenland ice core NGRIP2^50^; (**b**) Pb and Sb concentrations in Alpine ice cores^52^; (**c**,**d**) Pb/Al ratio in sediments of Lake Zoñar and Alboran Sea cores^33^; (**e**) Charcoal content in sediments (fragments/cm^3^) of Laguna de la Mula^36^; (**f**) Reconstructed d-excess and δ^18^O of evaporated dripwater in Sima Blanca Cave (this study). Major historical events related to the Roman Empire are indicated, including the occupation and abandonment of the Iberian Peninsula.

As a consequence of the Roman activity, the IRHP was characterized by an important increase in environmental pollution (Fig. 7). For example, there was an increase in heavy metal input to aquatic systems in both south and north Iberia^33,43,46–48^, as well as significant landscape modification^45^ during this period. Lead pollution from Roman mining in Iberia is recorded by lake sediment cores in Europe^49^ and ice cores in Greenland^50^, the Russian Arctic^51^ and the Alps^52^. Increased charcoal abundance in several lake sediment cores suggest intense deforestation and biomass burning during the early and middle-IRHP^36,38,48,53^ (Fig. 7).

Wetter conditions also prevailed in the western Mediterranean region at the time of the IRHP, as evidenced by lake records and a carbonate speleothem^20,54–57^. Moreover, increased rainfall during this period has been inferred from tree-ring growth in central Europe^58^ and analysis of carbon isotopes in ancient crop grains^59^, which suggest wetter climate conditions during the conquest and apogee of the Roman Empire in Iberia.

The SBL record suggests that from ~ 100 BCE until ~ 500–600 CE, a general aridification trend occurred that was punctuated by some brief wetter periods. Roman mining and intense land-use change declined in Iberia during this period^36,38,53^ until the Roman Empire eventually abandoned the Iberian Peninsula at ~ 400 CE. Indeed, a gradual decrease in mining and lead emissions occurred from ~ 100 to 250 CE, just before the Imperial Crisis (234–284 CE)^51^ (Fig. 7).

The Imperial Crisis has been attributed to a combination of migrations into the Roman territory, Barbarian invasions and civil wars, peasant rebellions, political instability, debasement of currency, and economic depression. Natural disasters, such as plague (i.e. the Plague of Cyprian, 249–262 CE) and/or increased climate variability, may have contributed to the fall of the Roman Empire^60^. Indeed, exceptional climate variability in Europe from ~ 250 to 550 CE is evidenced by tree-ring records, including periods of severe and prolonged droughts^58,61^. This was a time of increased aridification of SE Iberia. Drier winters, as suggested by the SBL record, probably resulted in crop failure and more extreme weather events that led to agricultural instability during the abandonment of Iberia by the Romans^17^.

The driest conditions inferred from the SBL stalactite occur at the transition between the IRHP and the Migration Period, at 500–600 CE. Cold and dry conditions occurred in the northern hemisphere during the Late Antique Little Ice Age (between 536 and 660 CE) and had an important impact on the transformation of ancient civilizations. Written sources pointing to significant veiling of solar radiation in 536 and 537 CE have been attributed to a powerful volcanic eruption that resulted in crop failures in different areas of the Roman Empire^61^. These events coincided with decreasing total solar irradiance in the Northern Hemisphere^62^ and an event of ice-rafting in the subpolar North Atlantic (cold event 1, 800 CE)^63^.

In summary, we demonstrate for the first time that stable isotopes of hydration water in gypsum speleothems can be used as a proxy for paleo-humidity/aridity. This novel approach offers the possibility of estimating all the oxygen (^16^O, ^17^O, ^18^O) and hydrogen (^1^H, ^2^H) stable isotopes of cave dripwaters as a proxy for evaporation. We suggest that gypsum speleothems in arid or semiarid regions constitute a valuable and unexploited archive of paleoclimate information.

## Methods

### Sample description

SBL is a 63-cm long gypsum stalactite that was naturally detached from the ceiling of Sima Blanca Cave when collected at a depth of ~ 10 m in 2007. The diameter of the SBL stalactite is relatively constant and ranges from 3 cm at the top (stratigraphically older) to 1.2 cm at the apex (stratigraphically younger). This indicates that the SBL was fed by a single infiltration-discharge point in the ceiling and preferentially grew in length, with minor lateral contributions. The structure of the SBL stalactite shows an inner feeding channel, ~ 0.5 cm wide, that conducted water from the feeding point to the stalactite’s tip, where drops of water evaporated to form gypsum. This conduit is similar to that found in carbonate stalactites. The SBL’s inner channel is partially filled with gypsum that probably precipitated during the last stage of formation of the speleothem before growth stopped, related to a reduction in water infiltration to the cave or a change in the water path in the epikarst. This inner part was avoided for sampling (Supplementary Fig. S2 online). The speleothem was cut along its longitudinal axis and we sampled concentric layers at constant distance of ~ 5 mm from the inner orifice. Five subsamples (3–10 g each) for U/Th dating were extracted along the main growth axis of the stalactite using a Dremel drill with 1 mm diameter bit (Supplementary Table S1 online and Fig. 2). Each of the sampling spots for both U/Th analyses represents 1–3 cm in depth. In addition, 28 subsamples of 150 mg every 2.3 cm were taken for stable isotope analysis of GHW and each sample spot is 0.5 cm wide. Because of the SBL stalactite grew mostly in length, the sampling along the main axis follows a sedimentary sequence, with the older gypsum samples closer the stalactite’s top and the younger samples at the top. No age reversals have been observed and the stalactite growth was apparently linear, suggesting that the sedimentary sequence has not been affected because of post-depositional processes.

### U-Th dating

U/Th dating analyses were carried out at the GEOTOP research centre facilities of the University of Quebec at Montreal (Canada). Powdered gypsum subsample (3–10 g) were weighed and transferred to 250 mL Teflon beakers, in which weighed amounts of a mixed spike ^233^U–^236^U–^229^Th had been placed and evaporated. The sample and spike were covered with water and transferred to a magnetic stirring hot plate and hot diluted HCl (~ 0.5 M) was added slowly until all the gypsum sample was completely dissolved. After the dissolution of the gypsum, ~ 7 mg of iron carrier (FeCl_3_) was added and the solution was left on the stirring plate overnight for spike sample equilibration. The uranium and thorium were separated from the bulk of the material with Fe(OH)_3_ precipitation using NH_4_OH. The precipitates were washed with MilliQ water and then dissolved in 6 N HCl. The U-Th separation was conducted using AG1X8 anionic resin bed, following the method in Ref.^64^. The U and Th fractions were deposited on Re filament between two layers of graphite and measured using a Triton Plus mass spectrometer (TIMS) equipped with an RPQ (retarding potential quadrupole)^65^. Mass fractionation for U was corrected by the double spike ^236^U/^233^U (1.1322), while mass fractionation for Th was considered negligible with respect to analytical error^65^. The overall analytical reproducibility, as estimated from replicate measurements of standards, is usually better than 0.5% for U concentration and ^234^U/^238^U ratios, and ranges from 0.5 to 1% for ^230^Th/^234^U ratios (2σ error range). The presence of non-authigenic ^232^Th is indicated by ^230^Th/^232^Th < 20 and implies the need for correction. We use a mathematical correction in which the U–Th isotopic composition of the detrital contamination is arbitrary estimated, in a manner resembling the approach in Ref.^65^. ^232^Th uses a correction and assumed a typical crustal Th/U ratio of 3.5, with ^234^U/^238^U and ^230^Th/^238^U activity ratios near secular equilibrium. In our model, we used ^232^Th/^238^U = 1.21 ± 50%, ^234^U/^238^U = 1 ± 10% and ^230^Th/^238^U = 1 ± 10%. Corrected ^230^Th/^238^U and ^234^U/^238^U activity ratios were then used to calculate the corrected ages (Supplementary Table S1 online). Comparisons between the uncorrected and corrected ages indicate that this adjustment has little effect on the ages for our samples (the magnitude of the correction being within the analytical errors). U–Th ages were calculated from the isotopic ratios ^235^U/^236^U, ^235^U/^234^U, ^236^U/^234^U, ^232^Th/^229^Th and ^229^Th/^230^Th using ISOPLOT/Ex version 2.0 software^66^. We use the age-depth modelling program COPRA^21^ to create the SBL chronology with proxy errors propagated to the temporal axis. The 95% uncertainties are based on 2,000-member ensembles of the age-depth relationship using *pchip* interpolation.

### Stable isotopes in gypsum hydration water (GHW)

Powdered gypsum samples were dried in an oven overnight at 45 °C. GHW was extracted by slowly heating each sample (~ 150 mg) to 400 °C, *in vacuo.* We used a bespoke offline extraction system consisting of six vacuum lines contained within a modified gas chromatography oven, following the methods in Ref.^10^. Oxygen (δ^18^O and δ^17^O) and hydrogen (δD) isotopes of GHW were measured simultaneously by cavity ring down spectroscopy (CRDS) in the Godwin Laboratory at the University of Cambridge (United Kingdom) using a L2140-i Picarro water isotope analyzer^24^. The results were normalized to the V-SMOW-SLAP scale by analyzing internal standards before and after each set of 12–15 samples. Four internal water standards (JRW, BOTTY, SPIT and ENR-15) were calibrated against V-SMOW and SLAP, using δ^17^O of 0.0‰ and − 29.69865‰, respectively, and δ^18^O of 0.0‰ and − 55.5‰, respectively^67^. This standardization considers ^17^O_excess_ = 0 for both international standards, where ^17^O_excess_ = ln (δ^18^O/1,000 + 1) − 0.528 × ln (δ^17^O/1,000 + 1)^68^. δD was calibrated against V-SMOW, GISP and SLAP. All isotopic deviations are reported in parts per thousand (‰) relative to V-SMOW and ^17^O_excess_ values are given in per meg units (0.001‰). External error (1SD) of the method was ± 0.05‰ for δ^17^O, ± 0.1‰ for δ^18^O, ± 0.7‰ for δD, ± 0.8‰ for d-excess (δD-8* δ^18^O) and ± 11 per meg (± 0.011‰) for ^17^O_excess_, as estimated by repeated analysis (n = 5) of an analytical grade standard, extracted together with five samples in each run of the extraction apparatus^10^.

## Supplementary information

Supplementary Information.
