# Author Correction: The potential of gypsum speleothems for paleoclimatology: application to the Iberian Roman Humid Period

**DOI:** 10.1038/s41598-020-74290-8

**Published:** 2020-10-09

**Authors:** Fernando Gázquez, Thomas K. Bauska, Laia Comas‑Bru, Bassam Ghaleb, José‑María Calaforra, David A. Hodell

**Affiliations:** 1 Water Resources and Environmental Geology Research Group, Department of Biology and Geology, University of Almería, Crta. Sacramento s/n, La Cañada de San Urbano, 04120 Almería, Spain; 2 Andalusian Centre for the Monitoring and Assessment of Global Change (CAESCG), University of Almería, Crta. Sacramento s/n, La Cañada de San Urbano, 04120 Almería, Spain; 3 Godwin Laboratory for Palaeoclimate Research, Department of Earth Sciences, University of Cambridge, Downing Street, Cambridge, CB2 3EQ UK; 4 British Antarctic Survey, High Cross, Madingley Road, Cambridge, CB3 0ET UK; 5 School of Archaeology, Geography & Environmental Sciences, University of Reading, Whiteknights, Reading, Berkshire, RG6 6DR UK; 6 Centre de Recherche en Géochimie Et Géodynamique (GÉOTOP-UQAM)-McGill University, 201 ave. du Président-Kennedy 7e étage, local PK-7150, Montréal, QC H2X 3Y7 Canada

Correction to: *Scientific Reports*, 10.1038/s41598-020-71679-3, published online 09 September 2020

The original version of this Article contained an error in the title of the paper, where the word “Humid” was incorrectly given as “Human”. This has now been corrected in the PDF and HTML versions of the Article, and in the accompanying Supplementary Information file.

