# Supplementary material for: The potential of gypsum speleothems for paleoclimatology: application to the Iberian Roman Human Period
Source: Sci Rep. 2020 Sep 9;10:14705. doi: 10.1038/s41598-020-71679-3 (PMC7481774; doi:10.1038/s41598-020-71679-3)
Supplement: Supplementary file 1 — Supplementary Information. [file 41598_2020_71679_MOESM1_ESM.pdf]

Supplementary material:

The potential of gypsum speleothems for paleoclimatology: Application to the Iberian Roman Human Period

Fernando Gázquez[1,2]*, Thomas K. Bauska[3,4], Laia Comas-Bru[5], Bassam Ghaleb[6], José-María Calaforra[1,2] and David A. Hodell[3]

[1]Water Resources and Environmental Geology Research Group. Department of Biology and Geology. University of Almería, Crta. Sacramento s/n, 04120 La Cañada de San Urbano, Almería, Spain.
[2]Andalusian Centre for the Monitoring and Assessment of Global Change (CAESCG), Crta. Sacramento s/n, 04120 La Cañada de San Urbano, University of Almería, Spain
[3]Godwin Laboratory for Palaeoclimate Research. Department of Earth Sciences. University of Cambridge. Downing Street, Cambridge, CB2 3EQ, United Kingdom.
[4]British Antarctic Survey, High Cross, Madingley Road, Cambridge, CB3 0ET, United Kingdom.
[5]School of Archaeology, Geography & Environmental Sciences, University of Reading, Whiteknights, RG6 6AB, United Kingdom.
[6]Centre de Recherche en Géochimie et Géodynamique (GÉOTOP-UQAM)-McGill University, Montreal, 201 ave. du Président-Kennedy 7e étage, local PK-7150, Montréal QC, H2X 3Y7, Canada.

*corresponding author (f.gazquez@ual.es)

1. Geological setting and cave description

Sima Blanca cave (37º07´36´´N; 2º03´21´´E) is located in the north-eastern sector of the gypsum karst of Sorbas (Almeria, SE Spain), a karstified 120-m thick sedimentary sequence of Messinian age (Yesares Member), consisting of alternating gypsum and carbonate marl (Dronkert, 1978; Evans et al., 2015). The

main passage of the cave (Maravillas Chamber) is ~15 m long and ~1.5 m high and developed in the uppermost marl stratum of the series, sandwiched between two gypsum strata ~10 m tick. The access to the cave is through a ca. 10 m deep shaft generated by dissolution of the upper gypsum stratum. The lower gypsum bed displays two vertical shafts, ca. 10 m in depth, with no man-size connection to deeper cave levels, unlike other cave systems in the karst of Sorbas (Gázquez et al., 2016; 2017a). The cave ceiling displays whitish gypsum speleothems, mostly stalactites and coralloids, whereas no stalagmites can be found on the cave floor. The cave lacks any permanent watercourse; drip points are scarce and generally inactive most of the year.

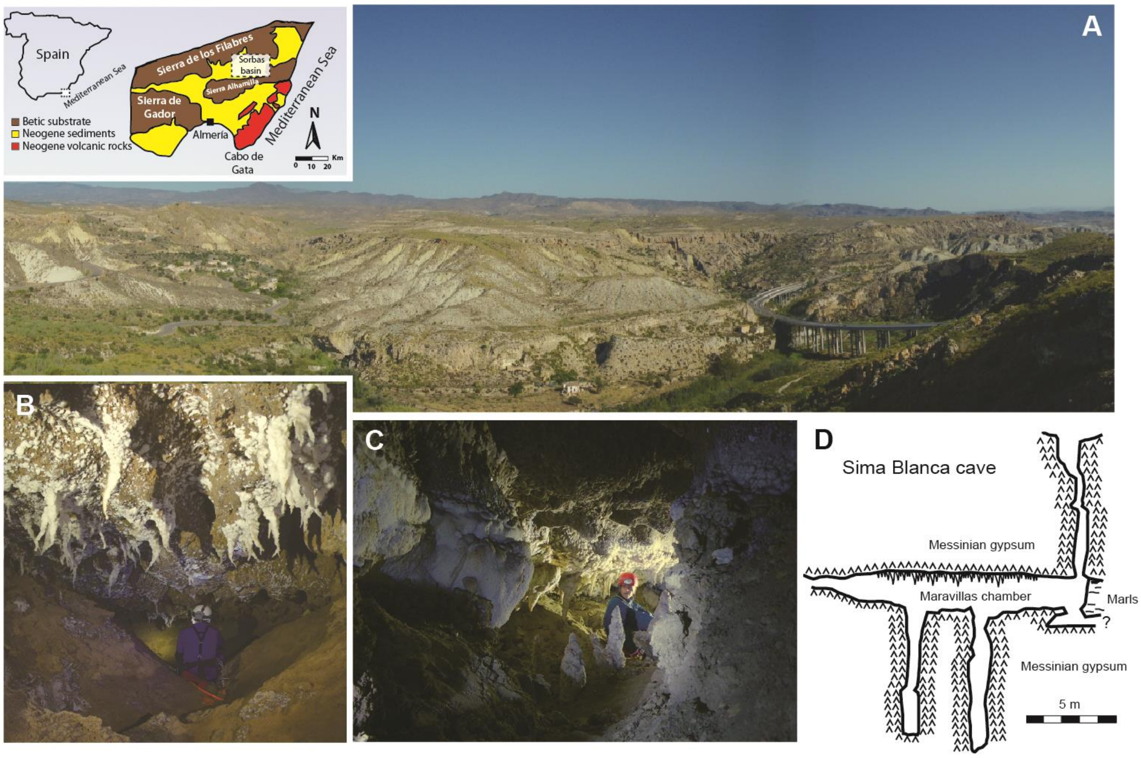

Supplementary Figure 1. Location of Sima Blanca Cave: A. Overview of the gypsum karst of Sorbas; B. Gypsum stalagmites in Sima Blanca Cave, C. Gypsum speleothem in Covadura Cave; D. Profile of Sima Blanca Cave.

Air circulation inside the Sorbas karstic network is complex and driven by thermal disequilibrium between surface and in-cave air, as observed in previous studies on the Covadura Cave System, 2 km apart from Sima Blanca and the same depth (~10 m). The mean annual temperature in the shallower galleries in the gypsum karst of Sorbas is 15.1±2.5ºC (Gázquez et al., 2017a) and relative humidity shows

a seasonal pattern, with relatively constant values close to 100% between May and December and lower and more variable between January and April (91±5%). The relative humidity values in these caves can be as low as ~65% during several consecutive days in winter (December to February) (Gázquez et al., 2017a).

The regional climate is semi-arid, with mean annual temperature of 18ºC (mean minimum of 11ºC in January and mean maximum of 30ºC in July) and a mean annual rainfall of 210 mm (minimum monthly mean in July and maximum in November). The mean annual relative humidity outside is 68±19% and occasionally can be <30% (Gázquez et al., 2017a). Some 80% annual rain falls during low-frequency rainstorm events, usually in the autumn (Esteban-Parra et al., 1998). This climate pattern is typical of the Mediterranean coast of the Iberian Peninsula, which is periodically affected by Isolated Depression at High Level (IDHL) systems (Milán et al., 1995). The estimated annual potential evapotranspiration for Sorbas is 1190 mm yr^{-1} (John and Harris, 2011), nearly five times the mean annual precipitation.

2. Stalactite inner structure

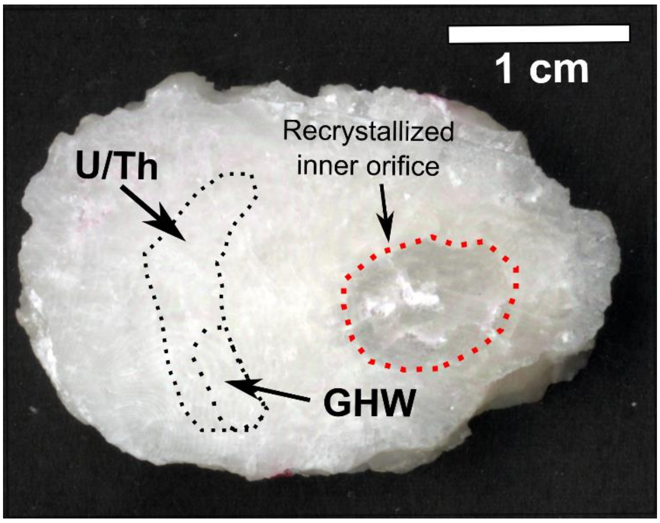

Supplementary Figure 2. Cross-section of the SBL stalactite at 9 cm from the top. The sampling positions for U-Th sample (i.e. SBL-09) and gypsum hydration water (GHW) sample (i.e. SBL-02B) are indicated. Note that the inner orifice of the stalactite is filled with gypsum that is interpreted as being post-depositional and was avoided for sampling.

3. U/Th dating results

Sample	Distance (cm)	Weight (g)	^{238}U (ppb)[a] ±2σ	^{232}Th (ppt) ±2σ	$^{230}Th/^{232}Th$ ±2σ	$^{234}U/^{238}U$ ±2σ	$^{230}Th/^{238}U$ ±2σ	^{230}Th Age uncorrected (years) ±2σ	^{230}Th Age Corrected (years) ±2σ
SBL-00	1.5±0.5	3.5	93.9±0.4	673±4	11.1±1.2	0.998±0.006	0.026±0.003	2865±313	2663±334
SBL-09	9±0.5	3.2	145.3±3.2	791±4	14±0.7	1.017±0.010	0.025±0.001	2756±145	2601±169
SBL-33	33±1.5	9.1	178±0.7	BQL	-	1.056±0.009	-	-	-
SBL-46	46±1.5	10.1	150±0.7	1201±5	6.6±0.2	1.029±0.011	0.017±0.000	1842±55	1585±136
SBL-61	61±0.5	3.4	131±0.4	1065±6	5.1±0.2	0.999±0.007	0.014±0.007	1489±66	1248±149

Supplementary Table 1. U-Th results and calculated ages of the SBL stalactite. "years BP" where BP is 1950 CE. We use a mathematical correction in which the U-Th isotopic composition of the detrital contamination is arbitrary estimated, in a manner resembling the approach by Ludwig and Paces (2002). ^{232}Th used as index for the correction and assumed a typical crustal Th/U ratio of 3.5 [atomic ratio], with $^{234}U/^{238}U$ and $^{230}Th/^{238}U$ activity ratios near secular equilibrium. In our model, we specifically used the initial activities ratios $^{232}Th/^{238}U$=1.21 ± 50%, $^{234}U/^{238}U$=1 ± 10% and $^{230}Th/^{238}U$=1 ± 10%. BQL: Below quantification limits.

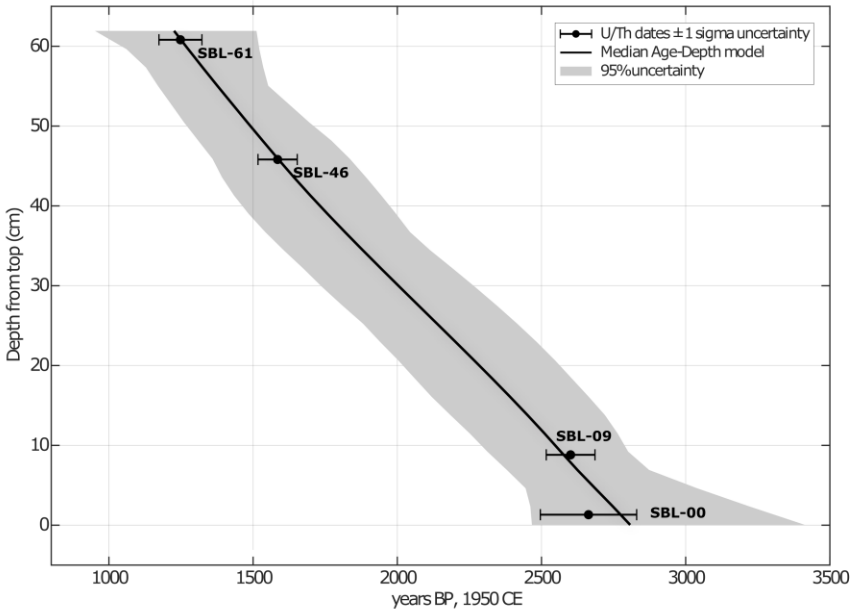

Supplementary Figure 3. Plot of age vs. distance from the ceiling of SBL gypsum stalactite. The COPRA-based age-depth model is anchored by four U-Th ages and indicates a near linear growth rate of 0.42 mm/year.

4. Stable isotopes of gypsum hydration water

Sample	Distance from the top (cm)	Age (years BP)	$\delta^{17}O_{ghw}$ (‰)	$\delta^{18}O_{ghw}$ (‰)	δD_{ghw} (‰)	$\delta^{17}O_{DW}$ (‰)	$\delta^{18}O_{DW}$ (‰)	δD_{DW} (‰)	d-excess$_{DW}$ (‰)	^{17}Oexcess$_{DW}$ (per meg)	H_2O (%)
SBL-1A	0	2804 ± 278	3.01 ± 0.04	5.73 ± 0.06	-25.01 ± 0.21	1.13	2.17	-4.81	-22.2	-18 ± 10	20.1
SBL-1B	2.3	2746 ± 213	2.85 ± 0.01	5.41 ± 0.03	-27.11 ± 0.06	0.96	1.85	-6.95	-21.7	-10 ± 15	20.5
SBL-1C	4.6	2685 ± 167	2.76 ± 0.03	5.25 ± 0.05	-26.84 ± 0.14	0.88	1.69	-6.67	-20.2	-16 ± 17	20.8
SBL-2A	6.9	2624 ± 149	2.43 ± 0.03	4.63 ± 0.04	-27.82 ± 0.24	0.55	1.08	-7.68	-16.3	-19 ± 13	20.9
SBL-2B	9.2	2568 ± 155	2.83 ± 0.03	5.42 ± 0.04	-26.50 ± 0.17	0.95	1.86	-6.32	-21.2	-30 ± 8	21.0
SBL-2C	11.5	2511 ± 165	2.62 ± 0.02	5.02 ± 0.03	-27.41 ± 0.17	0.74	1.46	-7.26	-18.9	-34 ± 18	20.8
SBL-3	13.8	2453 ± 172	2.77 ± 0.08	5.31 ± 0.11	-26.86 ± 0.41	0.89	1.75	-6.69	-20.7	-37 ± 7	20.3
SBL-4A	16.1	2393 ± 178	2.14 ± 0.02	4.12 ± 0.03	-28.95 ± 0.16	0.26	0.57	-8.83	-13.4	-38 ± 15	20.6
SBL-4B	18.4	2331 ± 178	2.17 ± 0.03	4.17 ± 0.04	-28.87 ± 0.25	0.29	0.62	-8.75	-13.7	-41 ± 16	20.4
SBL-5A	20.7	2268 ± 176	2.18 ± 0.03	4.19 ± 0.04	-28.87 ± 0.38	0.30	0.63	-8.75	-13.8	-36 ± 14	20.3
SBL-5B	23	2204 ± 173	2.40 ± 0.03	4.58 ± 0.04	-28.00 ± 0.18	0.52	1.02	-7.86	-16.0	-17 ± 10	20.8
SBL-6A	25.3	2139 ± 170	2.37 ± 0.03	4.52 ± 0.08	-28.83 ± 0.48	0.49	0.96	-8.71	-16.4	-15 ± 17	20.5
SBL-6B	27.6	2075 ± 166	2.29 ± 0.02	4.38 ± 0.04	-30.75 ± 0.39	0.41	0.82	-10.67	-17.2	-28 ± 13	20.6
SBL-6C	29.9	2010 ± 161	2.47 ± 0.07	4.73 ± 0.13	-29.37 ± 0.58	0.59	1.17	-9.26	-18.7	-31 ± 12	20.8
SBL-7	32.2	1946 ± 157	2.67 ± 0.03	5.12 ± 0.07	-26.66 ± 0.24	0.79	1.56	-6.50	-19.0	-37 ± 17	20.3
SBL-8A	34.5	1882 ± 156	2.51 ± 0.03	4.83 ± 0.03	-29.47 ± 0.09	0.63	1.28	-9.36	-19.6	-41 ± 13	20.6
SBL-8B	36.8	1819 ± 155	2.68 ± 0.01	5.12 ± 0.03	-27.22 ± 0.17	0.79	1.56	-7.06	-19.5	-29 ± 10	20.4
SBL-9A	39.1	1758 ± 153	2.55 ± 0.03	4.88 ± 0.08	-25.32 ± 0.47	0.67	1.32	-5.12	-15.7	-29 ± 14	20.8
SBL-9B	41.4	1699 ± 149	2.72 ± 0.02	5.22 ± 0.02	-26.02 ± 0.26	0.84	1.66	-5.83	-19.1	-33 ± 16	20.4
SBL-10A	43.7	1641 ± 143	2.89 ± 0.04	5.51 ± 0.04	-26.45 ± 0.44	1.01	1.95	-6.28	-21.9	-20 ± 11	20.3
SBL-10B	46	1568 ± 133	2.70 ± 0.04	5.16 ± 0.06	-27.40 ± 0.30	0.81	1.60	-7.25	-20.1	-31 ± 15	20.8
SBL-11A	48.3	1533 ± 121	2.55 ± 0.01	4.91 ± 0.03	-27.97 ± 0.21	0.66	1.35	-7.83	-18.6	-47 ± 18	20.5
SBL-11B	50.6	1480 ± 110	2.40 ± 0.02	4.63 ± 0.03	-29.30 ± 0.13	0.52	1.07	-9.19	-17.7	-46 ± 13	19.9
SBL-13A	55.2	1375 ± 101	3.03 ± 0.03	5.83 ± 0.04	-24.28 ± 0.09	1.15	2.27	-4.07	-22.2	-51 ± 12	20.1
SBL-13B	57.5	1324 ± 112	3.12 ± 0.03	6.00 ± 0.03	-23.66 ± 0.25	1.24	2.44	-3.43	-22.9	-49 ± 7	20.5
SBL-14A	59.8	1274 ± 134	2.63 ± 0.02	5.05 ± 0.03	-27.29 ± 0.15	0.75	1.49	-7.14	-19.0	-34 ± 11	20.6
SBL-14B	62.1	1224 ± 162	2.70 ± 0.02	5.16 ± 0.02	-26.68 ± 0.11	0.82	1.60	-6.51	-19.3	-25 ± 10	20.9

Supplementary Table 2. Oxygen and hydrogen stable isotopes (±1SD) in gypsum hydration water (GHW) in the SBL stalactite. Reconstructed paleo-dripwater values (DW) from GHW were calculated using fractionation factors by Gázquez et al., (2017b) and Liu et al., 2019. The U-Th age uncertainties are given as 95% CL and were calculated for each sample using COPRA code (Breitenbach et al., 2012).

References

Breitenbach, S. F. M., Rehfeld, K., Goswami, B., Baldini, J. U. L., Ridley, H. E., Kennett, D. J., Prufer, K. M., Aquino, V. V., Asmerom, Y., Polyak, V. J., Cheng, H., Kurths, J., Marwan, N. 2012. COnstructing Proxy Records from Age models (COPRA), Clim. Past, 8, 1765-1779.

Dronkert, H., 1978. Late Miocene evaporites in the Sorbas basin and adjoining areas. Memoria Societâ Geologica Italiana, 16, 341-362.

Evans N. P., Turchyn A. V., Gázquez F., Bontognali T. R. R., Chapman H. J. and Hodell D. A., 2015. Coupled measurements of $\delta^{18}O$ and δD of hydration water and salinity of fluid inclusions in gypsum from the Messinian Yesares Member, Sorbas Basin (SE Spain). Earth Planet. Sci. Lett. 430, 499–510.

Esteban-Parra, M.J., Rodrigo, F.S., Castro-Diez, Y., 1998. Spatial and temporal patterns of precipitation in Spain for the period 1880–1992. Int. J. Climatol. 18, 1557–1574.

Gázquez, F., Calaforra, J.M., Forti, P., De Waele, J., Sanna, L., 2015a. The role of condensation in the evolution of dissolutional forms in gypsum caves: study case in the karst of Sorbas (SE Spain). Geomorphology 229, 100–111.

Gázquez, F., Calaforra, J. M., Evans, N. P., Hodell D. A., 2017a. Using stable isotopes ($\delta^{17}O$, $\delta^{18}O$ and δD) of gypsum hydration water to ascertain the role of water condensation in the formation of subaerial gypsum speleothems. Chem. Geol. 452, 34–46.

Gázquez F., Evans N. P., Hodell D. A., 2017b. Precise and accurate isotope fractionation factors ($\alpha^{17}O$, $\alpha^{18}O$ and αD) for water and $CaSO_4 \cdot 2H_2O$ (gypsum). Geochim. Cosmochim. Acta, 198, 259–270.

Jones, P., Harris, I., 2011. Climate data provided by CRU TS 3.1 — University of East Anglia Climate Research Unit (CRU). CRU Time Series (TS) high resolution gridded datasets ([Internet]) NCAS British Atmospheric Data Centre, 2008 (accessed 15.01.10).

Ludwig, K.R., Pace, J.B. 2002. Uranium-series dating of pedogenic silica and carbonate, Crater Flat, Nevada. Geochim. Cosmochim. Acta 66 (3), 487-506.

Millán, M., Estrella, M.J., Caselles, V., 1995. Torrential precipitations on the Spanish east coast: the role of the Mediterranean sea-surface temperature. Atmos. Res. 36, 1–16.
